# An Account of a Remarkable Transposition of the Viscera in the Human Body

**Published:** 1789

**Authors:** Matthew Baillie


### VII. *An Account of a remarkable Transpofition of the Vifcera in the Human Body. By* Matthew Baillie, *M. B. From the Philofophical Tranfactions, Vol.* LXXVIII.; *with fome Alterations and Additions by the Author.*

*To* John Hunter, *Efq. F. R. S.*

Dear Sir,

A VERY fingular variety having occurred lately in the ftructure of the human body, I beg leave to communicate an account of it, by your means, to the Royal Society, if you fhould think it worthy of their notice. It happens by a very uncommon concurrence of circumftances, that while I am naturally led by the ties of affinity to apply to you upon this occafion, I can gratify my pride by thinking it is at the fame time to the perfon who is poffeffed of the firft reputation for his unwearied refearches in one of the moft extenfive, as well as interefting parts of the fyftem of nature.

I am, &c.

M. Baillie.

*Great Windmill Street,*
April 12, 1788.

There

There is nothing which tends more to illuſtrate the powers and the wiſdom of nature than the inveſtigation of the ſtructure of animals. We there find a moſt wonderful delicacy of mechaniſm, and exquiſitely adapted to a variety of purpoſes. This, however, is not to be better ſeen by following nature in her common tract, than by obſerving her wanderings. In theſe ſhe often ſhows more particularly the extent of her powers, and throws light on her ordinary plans. It is ſuch circumſtances which give importance or value to the obſervation of ſingular phænomena. The variety in animal ſtructure, an account of which I have the honour of preſenting to this learned Society, is a complete tranſpoſition, in the human ſubject, of the thoracic and abdominal viſcera to the oppoſite ſide from what is natural.

I have been at the pains to conſult many authors upon this ſubject, but with very little ſatisfaction. I ſhall not enter into a detail of what I have met with in the courſe of theſe reſearches; but ſhall briefly notice, that when any luſus of this ſort is mentioned, it is commonly in a ſingle ſentence or two, and the transſpoſition is not marked as univerſal *, or it is a change in the ſituation of ſome viſcus from diſeaſe. In ſhort, I have only found this ſingular luſus naturæ deſcribed by Cattierus, M. Mery, and M. Daubenton †; but by none of them is the deſcription ſufficiently particular. Enough has been ſaid to point out that they had met with exactly the ſame ſort of monſtroſity; but ſome circumſtances have been omitted, that I hope will be ſupplied by the preſent account,

* The partial tranſpoſitions to which I allude are of the heart. — See the Philoſophical Tranſactions for 1740-41, p. 777; Riolan. Animadverſ. in C. Bahiun, p. 703; Obſervata quædam Anatomico-Chirurgico-Medica rariora a D. C. E. Eſchenbach, p. 1; Hoffman Cardianaſtrophe, &c. 4to. Lipſiæ, 1671; Ephemer. Natur. Curioſ. Dec. 1. ann. 2, p. 139. —It is poſſible that there may alſo be other caſes upon record beſides what are here enumerated.

† See Cattieri Obſervat. 17, p. 49, apud Petri Borelli Centurias IV.; Mery's paper on this ſubject in the Hiſt. de l'Acad. Royale des Sciences, Tom. II.; and M. Daubenton's in the Deſcription du Cabinet du Roi, Tom. III. p. 204.

It may be obſerved that the caſe of Cattierus is mentioned by Th. Bartholin in centur. 2, obſervat. 29, p. 219; by Mentelius in the Epiſtolæ Gratulatoriæ apud Joan. Pecqueti Experimenta Nova Anatomica; and by Riolanus.

The count, which I proceed immediately to lay before the Society.

The perfon who is the fubject of this paper was a male, near forty years of age, fomewhat above the middle ftature, and of a clean active fhape. He was brought for diffection, in the common way, to Windmill Street. Upon opening the cavity of the thorax and abdomen, the different fituation of the vifcera was fo ftriking as immediately to excite the attention of the pupils who were engaged in diffecting it; and Mr. Cruikfhank, as well as myfelf, were very

The cafe of Mery is related in Nouveau Recueil d'Obfervations Chirurgicales par Monf. Saviard, p. 503, obfervat. 112; and of the fact defcribed by M. Daubenton, another account, by M. Sue, may be found in the Memoires des Scavans Etrangers, Tom. I. p. 292.

Since this paper was publifhed I have feen a fhort account of a tranfpofition of the vifcera in a clergyman, about thirty years of age, in the Philofophical Tranfactions, No. 107.

There is alfo an account of a tranfpofition of the vifcera in a boy of eighteen months, given by Caron in a periodical work by M. de Blegny, entitled "Le Temple d'Efculape, ou "le Depofitoire des nouvelles Decouvertes," printed at Paris in 1680. I have not feen the account myfelf; but it is alluded to in Haller's Bibliotheca Anatomica, Tom. I. p. 668, and in Lieutaud's Hiftoria Anatomico-Medica, Tom. I. p. 387.

foon informed of the fingularity. We were much furprifed as well as pleafed with the appearance; and I began immediately to examine every part of the change with confiderable attention: for this purpofe, after defiring a drawing to be made of the appearances as they were found upon opening the body, I next day injected it. The progrefs of the diffection has furnifhed various views, which are reprefented faithfully by drawings exhibiting the appearance of the change of fituation in all the individual vifcera and veffels *. I fhall not enter in my defcription into unneceffary minutiæ: this would render the paper lefs fuited to the Society, would not convey more information to perfons thoroughly acquainted with anatomy, and would rather tend to obfcure what is more important to thofe who have not given fo much attention to fubjects of this nature. It may not be improper to obferve, that, befides the tranfpofition in the vifcera of this perfon, there are feveral peculiarities which fometimes occur. I have taken notice of them in my defcription, although they are entirely independent of the tranfpofition.

* The drawings are in the poffeffion of the Author.

*Description of the Thorax.*

The mediaſtinum, or anterior duplicature of the pleura ſeparating the two cavities of the cheſt from each other, was found to incline obliquely downwards to the right ſide fully as much as it does commonly to the left ſide of the cheſt. The pericardium, too, inclined obliquely to the right ſide. On preſſing it gently away from the lungs, the phrenic nerves came diſtinctly into view, in their common ſituation; but the right phrenic nerve ran more obliquely, and was longer than the left. The lung, upon the right ſide, was divided by a ſingle oblique fiſſure into two lobes, having at the ſame time a deficiency oppoſite to the apex of the heart; and the lung on the left ſide was divided into three lobes, exactly contrary to what is found in ordinary caſes.

On opening the pericardium, the apex of the heart was found to point to the right, nearly oppoſite to the ſixth rib; and its cavities, as well as large veſſels, were completely tranſpoſed. What are commonly called the right auricle and ventricle were ſituated on the left ſide, and the left auricle and ventricle on the right. The pulmonary artery aſcended towards the right ſide of the the cheſt. The aorta was alſo directing its arch to the right; and the vena cava ſuperior, as well as inferior, were ſeen opening into their auricle on the left ſide of the ſpine.

On the outſide of the pericardium the tranſpoſition of the larger veſſels was very ſtriking. The longer ſubclavian vein was paſſing from the left ſide obliquely to the right before the branches which are ſent off from the arch of the aorta. The left carotid and ſubclavian arteries were found to ariſe from the arch of the aorta by one common trunk; the right carotid and ſubclavian to ariſe ſeparately.

In the duplicature of the pleura behind, or what may be called the poſterior mediaſtinum, there was a change correſponding to what we have already deſcribed. The deſcending aorta was found paſſing on the right ſide of the ſpine. The œſophagus was before it, inclining more and more to the right towards its lower extremity; and it at length perforated the diaphragm ſomewhat on the right ſide of the ſpine. The vena azygos was on the left ſide of the ſpine, opening in the common way into the vena cava ſuperior, which we formerly mentioned to be alſo tranſpoſed in its ſituation. The thoracic duct was ſeen in the middle between the de-

ſcending

ſcending aorta and the vena azygos, in ſome places forming a plexus of ſmall branches, in another dividing itſelf into two branches, which afterwards re-united in a common trunk, and at length climbing up to terminate in the angle between the jugular and ſubclavian veins on the right ſide of the body. The recurrent nerve of the par vagum, on the right ſide, paſſed round the beginning of the deſcending aorta, and, on the left, paſſed round the common trunk of the carotid and ſubclavian arteries. The large intercoſtal nerves being exactly under the ſame circumſtances on each ſide, it was impoſſible there could be any tranſpoſition in them. It appears, then, from the foregoing deſcription, that every thing admitting of ſuch a change was completely tranſpoſed in the thorax.

*Of the Abdomen.*

The liver was ſituated in the left hypochondriac region, the ſmall lobe being towards the right, and the great lobe in the left ſide. The ligaments uniting it to the diaphragm correſponded to this change, the right tranſverſe ligament being longer, and the left being ſhorter than uſual. The ſuſpenſory ligament could undergo little change, except that of being puſhed

 to to the left ſide along with the liver. On preſſing upwards the liver, ſo as to exhibit its poſterior and under ſurface, the gall bladder was ſeen on the left ſide preſerving its proper relative ſituation to the great lobe of the liver; and the veſſels of the portæ were found upon diſſection to be tranſpoſed, correſponding to the change of circumſtances. The hepatic artery was found climbing up obliquely from the right towards the left, before the lobulus Spigelii, and entered at the portæ into the ſubſtance of the liver by two or three branches on the right of the other veſſels. The ductus communis choledochus was on the left of the other veſſels, being formed from the ductus hepaticus and ductus cyſticus in the common way, and it paſſed obliquely downwards on the left, to terminate in the duodenum. What was moſt remarkable, of which indeed I never ſaw or heard of an inſtance before, it terminated in the fore part of the duodenum. The vena portarum paſſed behind the hepatic artery and ductus communis choledochus, aſcending obliquely towards the left ſide.

The ſpleen was ſituated in the right hypochondriac region, adhering to the diaphragm in the common way. What was very remarkable was, there being three ſpleens, nearly of the ſize of a pullet's egg, found adhering to the larger ſpleen by ſhort adheſions; beſides two other ſtill ſmaller ſpleens which were involved in the epiploon at the great end of the ſtomach. I never ſaw ſo many ſmall ſpleens in any one ſubject. The pancreas was found on the right ſide behind the ſtomach, running obliquely from the ſpleen to the curvature of the duodenum, and had its duct entering in common with the ductus communis choledochus into the cavity of that inteſtine. The ſplenic veſſels were paſſing along the upper edge of the pancreas to the right ſide, correſponding to the change of ſituation in the pancreas and ſpleen.

The ſtomach was ſituated on the right ſide, partly hid by the ſmall lobe of the liver, was paſſing to the left, and terminating in the pylorus ſomewhat on the left ſide of the ſpine. The duodenum took a moſt ſingular courſe; it paſſed to the right ſide, behind the ſmall end of the ſtomach; it then turned upon itſelf towards the left ſide; it afterwards took its proper ſweep to the right ſide, paſſing behind the ſuperior meſenteric artery and the greater meſaraic vein. The meſentery began to be formed on the right ſide inſtead of the left, as in ordinary caſes.

The ilium terminated in the great inteſtine on the left ſide, and there was in it a diverticulum of conſiderable ſize, a luſus not unfrequently occurring. The cæcum was ſituated on the left pſoas magnus and iliacus internus muſcles. The tranſverſe arch of the colon paſſed from the left to the right ſide of the body; and the ſigmoid flexure croſſed over the right pſoas to get into the cavity of the pelvis.

The kidnies had their veſſels tranſpoſed, as we ſhall remark more particularly afterwards. The renal capſules had undergone no change, as no variety could be produced by a tranſpoſition.

The aorta paſſed between the crura of the diaphragm into the cavity of the abdomen, and adhered, in its courſe, to the ſpine, on the right ſide of the vena cava inferior. Its branches were directed in their courſe, correſponding to the peculiar ſituation of the viſcera. The ſplenic and coronary arteries were paſſing to the right ſide, and the hepatic artery obliquely to the left. The ſuperior and inferior meſenteric arteries were directed to the right ſide. There was no change in the ſpermatic arteries; any tranſpoſition in the teſticles (if ſuch a thing could take place) not being capable of affecting them.

them. The lumbar arteries could alſo undergo little change, except that the left lumbar arteries muſt neceſſarily, from the peculiar ſituation of the aorta, be the longeſt. The vena cava inferior perforated the tendinous portion of the diaphragm, and adhered in its courſe to the ſpine on the left ſide cf the aorta.

The right emulgent vein was much longer than uſual, paſſing from the right kidney, before the aorta, to terminate in the vena cava inferior; and the left emulgent was much ſhorter, paſſing from the left kidney to the vena cava, which was ſituated on the left ſide of the ſpine. The right ſpermatic vein was found to open into the right emulgent, and the left into the vena cava inferior, about an inch under the left emulgent. The vena portarum was changed from its natural courſe, paſſing obliquely upwards to the left ſide, and its large branches, viz. the vena ſplenica, meſaraica major and minor, were all directed towards the right ſide of the ſpine.

There was no change in the intercoſtal nerve within the cavity of the abdomen; nor does it ſeem to be capable of being affected by any tranſpoſition of parts. We ſee, then, that there was a complete tranſpoſition of the abdominal viſcera, viſcera, each of them preſerving its proper relative ſituation to the others.

I examined the brain, the organs of ſenſe, of generation, the muſcles and blood veſſels of the extremities, but found nothing in them remarkable. Indeed I had no expectation of it; for all theſe parts are perfectly independent of thoracic or abdominal viſcera; but I did it to ſatisfy myſelf and the curioſity of others, who might wiſh to put ſuch a queſtion, or have ſuch a queſtion ariſing in their minds.

This perſon ſeems to have uſed his right hand in preference to his left, as is uſually the caſe, which was readily diſcovered by the greater bulk and hardneſs of that hand, as well as the greater fleſhineſs of the arm. It was not to be expected he ſhould be left-handed; but I mention this circumſtance too with a view to ſatisfy a curioſity which I know has been excited in many who have heard of this luſus.

I have been at conſiderable pains to learn ſomething of the hiſtory of this perſon during life; but the particulars I have heard are applicable only to the circumſtances of common men, having no connexion with ſingularity of ſtructure; and therefore, I think, it would be abuſing abuſing the time of the Society to give any account of them. One thing it may be right to mention is, that the perſon, while alive, was not conſcious of any uncommon ſituation of his heart; and that his brother, whom I have ſeen, has his heart pointing to the left ſide, as in ordinary caſes. Indeed there was little reaſon to expect that we ſhould meet with any thing particular in the account of his life. His health could not be affected by ſuch a change of ſituation in his viſcera; nor could there ariſe from it any peculiar ſymptoms of diſeaſe. Still leſs could there be any connexion between ſuch a change and his diſpoſitions or external actions. He might have known that his heart was directed towards the right ſide; but if we conſider how little every perſon, eſpecially thoſe of the lower claſs, are attentive to circumſtances not very palpable, it was ſcarcely to be expected he ſhould know it. If I had met with any thing in his life which was at all referable to the ſingularity of ſtructure, I ſhould have been very glad to have gratified the public curioſity by giving an account of it *.

Every

* Since the above luſus has occurred, I have ſeen, in the poſſeſſion of Mr. Payne, Surgeon, a fœtus, at the full time, with

Every fingular phænomenon in animal ftructure is worth remarking, even if it fhould not lead immediately to any ufeful obfervation; but it becomes more important if it fhould tend to throw any light upon the principles of nature in the formation of animals. It is reafonable to think that nature fhould follow fome general plan in her operations. There is fome effect which fhe has in view, and fhe will generally employ the fame means to produce it. In the ftructure of any animal, her view is to form fuch a combination of parts as to render the animal fitted for certain purpofes. She will commonly form the fame combination where the fame purpofes are to be ferved; or, in other words, there will be the fame ftructure in the fame fpecies of animals. The fame effect, however, may be produced, without a ftrict adherence to the employment of the fame means, as we find to be the cafe in all human inventions; and therefore there is no reafon why nature fhould not fometimes deviate from her orwith the vifcera tranfpofed. In the Anatomical Collection of Chrift Church, in Oxford, there is a heart tranfpofed that had belonged to a very fmall fœtus, but the fœtus itfelf is not preferved.

dinary plans. Accordingly we find there is much variety in animal ſtructure; but this does not commonly affect the animal functions. Under this reſtriction, the variety is ſo great in the appearances of every part of an animal, that it is almoſt impoſſible to examine any two animals of the ſame ſpecies without remarking many differences.

In the bony compages of an animal we find little variety in the extremities of bones where there is the apparatus of a joint, becauſe a particular ſhape is beſt adapted to a particular kind or latitude of motion. In other parts of the bones, where a difference of features is not material, there is great variety, as in the foramina, depreſſions, ridges, and ſutures of bones.

The ſame general rule will apply to variety in muſcles. The principal object is a certain inſertion near a joint, ſo as to give a determined direction of motion. With reſpect to ſuch inſertions, there is, comparatively ſpeaking, little variety; but there is a great difference in the bodies and connexions of muſcles, which have no ſhare in the regulation of the motion.

There is no part of an animal where there is a greater latitude of variety than in the diſtribution of blood veſſels. The reaſon of it is very obvious. The only object in the distribution of blood vessels is, to carry blood to every part of the body, and bring it back to the heart. The parts of an animal, in order to be supported, must be visited by successive changes of fresh blood; but it surely cannot be an object of importance whether the blood passes by one route or another. Hence the variety in blood vessels is extremely great. Still, however, there is a method in the deviations of nature, so that they may be marked or noted, the same varieties occurring in different animals.

It cannot be at all important to the function of a viscus whether it be in one mass or in separate portions; the structure being the same, the same action will take place: hence we often find the two kidnies joined together, forming one mass, and not unfrequently two or three spleens besides the common one. Neither can it be important whether a viscus should always be of the same shape, because its functions do not depend on shape, but on structure: we find accordingly, in this particular, much variety.

There are many of the viscera which are connected together in their functions, or by the junction of large blood vessels, in such a way as to require nearly the same relative situation

 among among themfelves. This becomes alfo neceffary in order to preferve the general fhape of the animal. Accordingly we find that when any important vifcus is changed in its fituation, it affects the fituation of other vifcera requiring in them a correfponding change. We faw in the perfon who is the fubject of this paper, that a change in the fituation of the heart and liver was accompanied with a change of fituation in the ftomach, fpleen, pancreas, and, in fhort, the whole abdominal vifcera. This, however, is a great deviation in nature; for it is nothing lefs than changing almoft the whole vital fyftem in an animal, and therefore it rarely happens.

In fuch a change, it does not appear that the functions can be affected, as they depend on ftructure and fituation, which are both preferved: hence the perfon who is the fubject of this paper arrived at the age of maturity, and might have continued to have lived to an extreme old age. The human machine might have been conftructed in this way generally; and, under fuch circumftances, what is now called the natural fituation of parts would have been as fingular as the prefent phænomenon.

There appears to be lefs variety in the nervous fyftem of animals of the fame fpecies than in moſt parts of the body. There is ſcarcely any difference in the appearance of the brain, and much leſs in the diſtribution of the nerves than of the blood veſſels. There is alſo little variety in the organs of ſenſe; perhaps the mechaniſm in both theſe is nicer, ſo that a conſiderable deviation would interfere with their peculiar functions.

The moſt common great deviations which nature produces in the ſtructure of an animal are various kinds of monſtroſity, by which the animal becomes often unfit for continuing its exiſtence. This ſort of imperfect formation, ſo much below the ſtandard of nature's common work, will have a tendency to check the propagation of great varieties, and thereby to preſerve an uniformity in the ſame ſpecies of animals.

It has been much agitated whether monſtroſities depend on the original formation, or are produced afterwards in the gradual evolution of an animal. This does not appear to be a queſtion of much importance; nor, perhaps, can it be abſolutely determined. But, upon the whole, it is more reaſonable to think that the ſame plan of formation is continued from the beginning, beginning, than that at any ſubſequent period there is a change in that plan.

It may be obſerved, that it is exactly the ſame creative action which produces the natural ſtructure, or any deviation from it; for, in caſes of deviation, the action is either carried too far, ceaſes too ſoon, or is diverted into uncommon channels. This will explain the various kinds of monſtroſity from redundancy, deficiency, or tranſpoſition of parts.

